# Relationships of Age and Sex with Cytokine Content and Distribution in Human Platelet Fibrin Gels

**DOI:** 10.1038/s41598-018-28376-z

**Published:** 2018-07-13

**Authors:** Meng-Yi Bai, Meng-Han Chuang, Ming-Fang Lin, Sung-Ling Tang, Chin-Chean Wong, Wing P. Chan

**Affiliations:** 1 Graduate Institute of Biomedical Engineering, National Taiwan University of Science and Technology, Taipei, 10607 Taiwan; 2 Biomedical Engineering Program, Graduate Institute of Applied Science and Technology, National Taiwan University of Science and Technology, Taipei, 10607 Taiwan; 3 Department of Radiology, Wan Fang Hospital, Taipei Medical University, Taipei, 11617 Taiwan; 4 Department of Medical Imaging and Radiological Technology, Yuanpei University, Hsinchu, 30015 Taiwan; 5 Department of Orthopedics, School of Medicine, College of Medicine, Taipei Medical University, Taipei, 11031 Taiwan; 6 Department of Orthopedics, Shuang Ho Hospital, Taipei Medical University, New Taipei City, 23561 Taiwan; 7 Department of Radiology, School of Medicine, College of Medicine, Taipei Medical University, Taipei, 110 Taiwan

## Abstract

We aimed to determine relationships between age and sex with cytokine content and distribution in human platelet-rich fibrin (PRF) gel. Rabbit PRF was harvested from whole blood (*n* = 6). Human PRF was collected from 36 healthy volunteers (1:1 men:women) without systemic diseases and not current undergoing medical treatment. Histological analysis and optical microscopy were used to assess the three-dimensional structure of the PRF network. Enzyme-linked immunosorbent assays, quantification of adenosine triphosphate, and bioluminescence imaging of PRF sections were used to assess cytokine and entrapped platelet distribution. Three-dimensional structures of fibrin networks revealed concentration gradients of the platelet-derived growth factor beta beta homodimer and the transforming growth factor-beta 1. Histological analysis of PRF sections (from the red blood cell end to the plasma end of a clot) showed a gradual increase in average porosity, most prominently in PRF clots from young and middle-aged men and women, and a decrease in compactness along the longitudinal axis of the PRF gel. The end of the PRF gel closest to the red blood cell layer is the essence of the PRF clot, and the ability to generate platelets depends on sex and age in humans.

## Introduction

Platelet-rich plasma (PRP) and platelet-rich fibrin (PRF) are innovative surgical materials used in tissue engineering applications^1^. Introduced in the 1970s and becoming increasingly popular since the 1990s^2^, PRP gels are derived from autologous blood, and platelets are the main constituent. Improvements in bone regeneration are brought about by the high concentrations of bioactive proteins in PRPs^3,4^. Originally shown to be effective in alveolar ridge augmentation, the technique has found applications in periodontal and oral maxillofacial surgery^5^. The mechanism of action is not well understood, but PRP gels are believed to provide high concentrations of growth factors, including tissue growth factor and platelet-derived growth factor (PDGF), which can mediate the proliferation of mesenchymal stem cells and increase collagen formation and matrix synthesis^6^. Although PRP has wide clinical applications in various healing therapies and specialties, such as orthopedics and ophthalmology, studies of the effects of PRP have provided inconsistent results. For example, although many *in vitro* studies have found several growth factors to enhance muscle regeneration, few *in vivo* studies have been performed, and no high-quality randomised trials of this treatment have been completed^7–9^. Systematic reviews and meta-analyses have found insufficient evidence to recommend PRP treatment for overuse tendinopathy^10^. Some researchers have attributed these inconsistencies to the very-short-term effects of grafted PRP, given the rapidly decreasing concentration of bioactive proteins^11^.

A self-clotted preparation of PRP derivatives, PRF gel, has overcome some of these hurdles. Choukroun’s PRF, a recently-developed second-generation platelet concentrate, was prepared without adding chemicals to blood samples before centrifugation^12,13^. The advantage of PRF is that no anticoagulants and thrombin additives are necessary. As a completely autologous fibrin matrix containing platelets and leukocyte growth factors, PRF can slowly release bioactive proteins (growth factors)^3,5,12^. The platelet (thrombocyte) count is three to seven times greater in PRF than in blood. The growth factors obtained from PRF include PDGF, transforming growth factor (TGF), and insulin-like growth factor (IGF)^2,5^. Several studies confirm that PDGF and TGF are gradually released from PRFs (28 days), whereas they are released within 1 day from PRPs^10,13^. A possible explanation is that PRF polymerises into a three-dimensional structure progressively, slowly and naturally during centrifugation, helping to entrap cytokines released from platelets within the fibrin network. In contrast, PRP is activated by concentrated thrombin, causing low-molecular-weight fibrin to rapidly polymerise and then strongly contract, expelling fluids. This process interferes with the entrapment of cytokines released from platelets and with the controlled release of cytokines from the fibrin network. Thus, the concentration of growth factors and the fibrin structure can strongly influence tissue regeneration.

In a previous study of PRF gels using a rabbit model, platelet cytokines, particularly the PDGF beta beta homodimer (PDGF-BB) and TGF-beta 1 (TGF-β1), formed concentration gradients where higher concentrations were at the red blood cell (RBC) end and lower concentrations were at the plasma end. Fibrin provides a matrix for the migration of fibroblasts and endothelial cells, both of which, because they are involved in angiogenesis, are responsible for healing^14^. Therefore, the characteristics of the three-dimensional fibrin network should be considered. In that animal model, histological analysis was used to reveal that the porosity in PRF samples gradually increased from 6.5% at the RBC end to 40.3% at the plasma end. These important findings can be used to improve the clinical efficacy of PRFs. Moreover, a previous report demonstrated that both age and sex influence the levels of some platelet cytokines in PRPs^15^, and this effect could account for inconsistencies between reports of the clinical benefits of PRP treatment. Thus, age and sex should be considered in any PRF investigation.

Based on the results of the rabbit model study, we launched a pilot clinical trial to ascertain whether other methods, such as a second centrifugation step, can be used to obtain more efficacious cytokine-fibrin networks for implantation from the original and entire PRF entity. We then used standard peripheral blood collection and centrifugation procedures to obtain PRF gels for examination via enzyme-linked immunosorbent assays (ELISAs), histology and optical microscopy. We verified our findings in humans via comparisons with previous findings in rabbits^16^. In this study, we used human PRF gels obtained from centrifuged whole blood to assess how the content and distribution of various cytokines are related to age, sex and the three-dimensional fibrin network structure of PRF. These results could improve the clinical applications of PRF and aid clinicians in determining whether PRF is clinically useful.

## Methods

### Preparation of PRF Gel and Matrix, Animal Model

For ethical reasons, the number of animals used was minimized yet was sufficient to obtain statistically valid results. The sample size per group was calculated based on an estimated between-group difference of 0.5, assuming a two-sided confidence interval of 95% and a variance of 0.5.

Six male New Zealand rabbits (7 to 51 weeks old weighing 2.7 to 3 kg) were used for PRF gel preparation. The PRF was prepared using the protocol described by Dohan *et al*.^17^ with modifications that improved reproducibility^16^. The PRF (a pale yellow elastic gel-like strip) was pre-frozen at −20 °C for 30 min and then transferred to another freezer for storage at −80 °C until the cytokines were quantified. The gradual freezing protocol was used to avoid cytokine protein denaturation; cytokine levels can be reduced by improper handling and storage^18^. Each of the six PRF samples was divided into three segments with equal length for cytokine quantification. Cytokine activation was conducted in 4-(2-hydroxyethyl)-1-piperazineethanesulfonic acid (≥99.5%, Sigma-Aldrich, St. Louis, MO, USA), pH-adjusted to 7.2–7.6 using sodium hydroxide (≥98%, Sigma-Aldrich) or hydrochloric acid (1 N, Sigma-Aldrich). Cytokine concentration was measured using sandwich-type ELISA kits (Nos. MBS165883, MBS165545 and MBS704445; MyBiosource, Inc., San Diego, CA, USA) according to manufacturer’s instructions. After the exudate was removed from the defrosted PRF gel, the remaining white PRF matrix was fixed in 10% formalin (Sigma-Aldrich) for 24 h and dehydrated in a series of ethanol solutions (starting at 70% and reaching 100%) for use as histological specimens. To obtain the cytokine extract, a second centrifugation process at 4000 rpm was applied to the freshly-prepared PRF gel for 5 min, after which time, the shrunken sponge-like aggregate was precipitated at the bottom of the Eppendorf tube. The supernatant, removed using a micropipette, was labeled extract1.

### Preparation of PRF Gel and Matrix, Human Model

All experiments using humans were approved by the Taipei Medical University - Joint Institutional Review Board (Certificate No. TMU201502016) according to the ethical standards of the responsible committee on human experimentation (institutional or regional) and the Helsinki Declaration of 1975, as revised in 1983. All volunteers received clear and honest information about the nature and objectives of the study before testing. Written informed consent was obtained from all participants. Blood samples were treated according to a previously published PRF protocol^16^. All methods were carried out according to relevant guidelines and regulations.

Whole blood was collected from 36 healthy volunteers (1:1 men:women) without systemic diseases or current medical treatments. Participants were colleagues and medical professionals who received annual health check-ups, and those of each sex were placed into three subgroups based on age: 20–39 years, 40–59 years and 60 years or more. Samples were then collected without anticoagulant in 10-mL glass-coated plastic tubes (Vacuette^®^) and were immediately centrifuged at 3000 rpm for 10 min in a DSC-200A-2 table top centrifuge (Digisystem; Laboratory 315 Instruments Inc., Taipei, Taiwan). Forceps were used to remove the freshly-generated PRF gels from the 10-mL glass-coated plastic tubes, and each of the thirty-six PRF samples was stored at −80 °C then divided into three equal segments for cytokine quantification.

### Cytokine Quantification and Histological and Optical Microscopic Analyses

Cytokines were quantitively measured using sandwich-type ELISA kits (Human PDGF-BB Quantikine ELISA Kit, Human TGF-beta 1 Quantikine ELISA Kit and Human IGF-I Quantikine ELISA Kit; bio-techne/R&D Systems, Minneapolis, MN, USA). All steps were performed according to manufacturer’s instructions. Results were obtained by first measuring absorbance at 450 nm then determining cytokine concentration against a standard curve.

Prior to histological analysis, each PRF sample was fixed in 10% formaldehyde, dehydrated, placed in a tissue cassette, and embedded in paraffin wax. The infiltration of paraffin into the porous tissue helped maintain the intrinsic morphology of the tissues and also solidified the sample, allowing thin sectioning. After dividing the samples into three equal segments, the blocks were cut into 3- to 5-μm sections for histological analyses. Tissue sections were mounted on slides, stained with hematoxylin-eosin or Masson’s trichrome and viewed and photographed under an optical microscope (DM 2000; LEICA, Wetzlar, Germany). The compactness of the fibrin network (i.e., porosity [*n*]) was recorded and measured directly in an optical microscope control computer using ImageJ software (NIH, Bethesda, MD, USA).

$${\rm{Porosity}}\,[{n}]={{\rm{A}}}_{{\rm{p}}}/{{\rm{A}}}_{{\rm{t}}}\times 100 \% ,$$

where A_p_ denotes the total measured area of the pores in each optical microscopic image, and A_t_ is the total area of the optical microscopic image. Fiber diameter was measured from the optical microscopic image using DiameterJ software (NIST, Gaithersburg, MD, USA), an open source plug-in created for ImageJ. DiameterJ uses a two-step process: (1) the image is segmented into a binary image (black and white pixels only) and (2) the segmented image is analysed.

### Statistical Analysis

All data generated or analysed are included in this published article. Cytokine concentrations and fibrin network porosities were determined for all harvested PRFs (rabbit and human). Student *t* tests were used to compare the results obtained for PRFs and sera (controls). Differences were recognised as statistically significant when *P* < 0.05. Excel (Microsoft, Seattle, WA, USA) was used for statistical analysis.

### Ethics approval and consent to participate

The animal breeding practices and animal use protocols were approved by the Institutional Animal Care and Use Committee (IACUC) of Taipei Medical University (Certificate No. LAC-2013-0072). All experiments on humans were approved by the Taipei Medical University-Joint Institutional Review Board (Certificate No. TMU201502016) in accordance with the ethical standards of the responsible committee on human experimentation (institutional or regional) and with the Helsinki Declaration of 1975, as revised in 1983.

## Results

### Quantification of Cytokines in Rabbit PRF Gels

**Figure 1:**
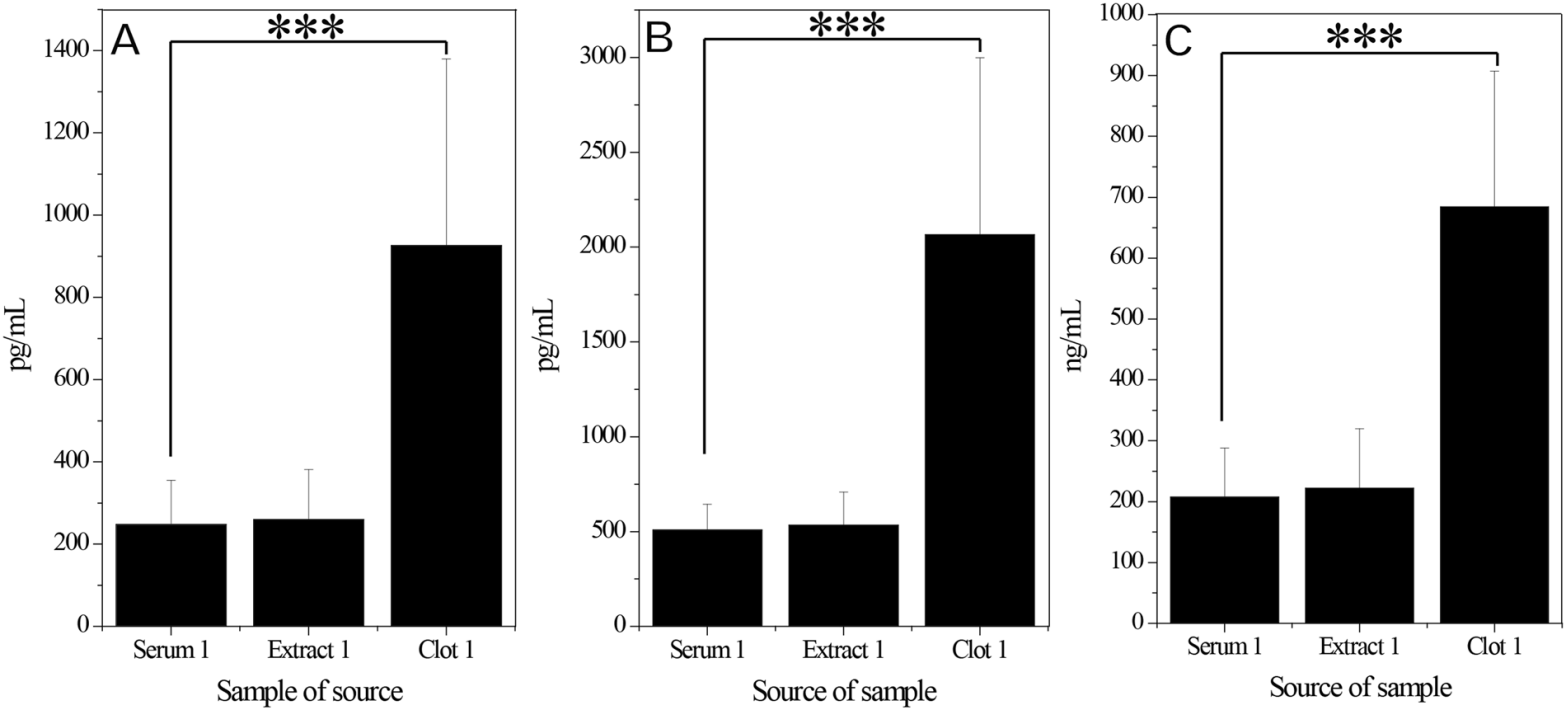
Rabbit study: Levels of cytokines in platelet-rich fibrin (PRF) gel. (**A**) Platelet-derived growth factor beta beta (PDGF-BB), (**B**) transforming growth factor beta-1 (TGF-β1) and (**C**) Insulin-like growth factor 1 (IGF-1). All values are presented as means ± standard deviations across six rabbits. The extrudate of the PRF close to the red blood cell end is denoted as clot1. The extrudate of the PRF gel after a second centrifugation is denoted as extract1. Serum was obtained from clotted whole blood after an initial centrifugation. Concentrations of PDGF-BB and TGF-β1 in clot1 were always three to four times higher than those in extract1, which were similar to those in serum.

**Figure 2:**
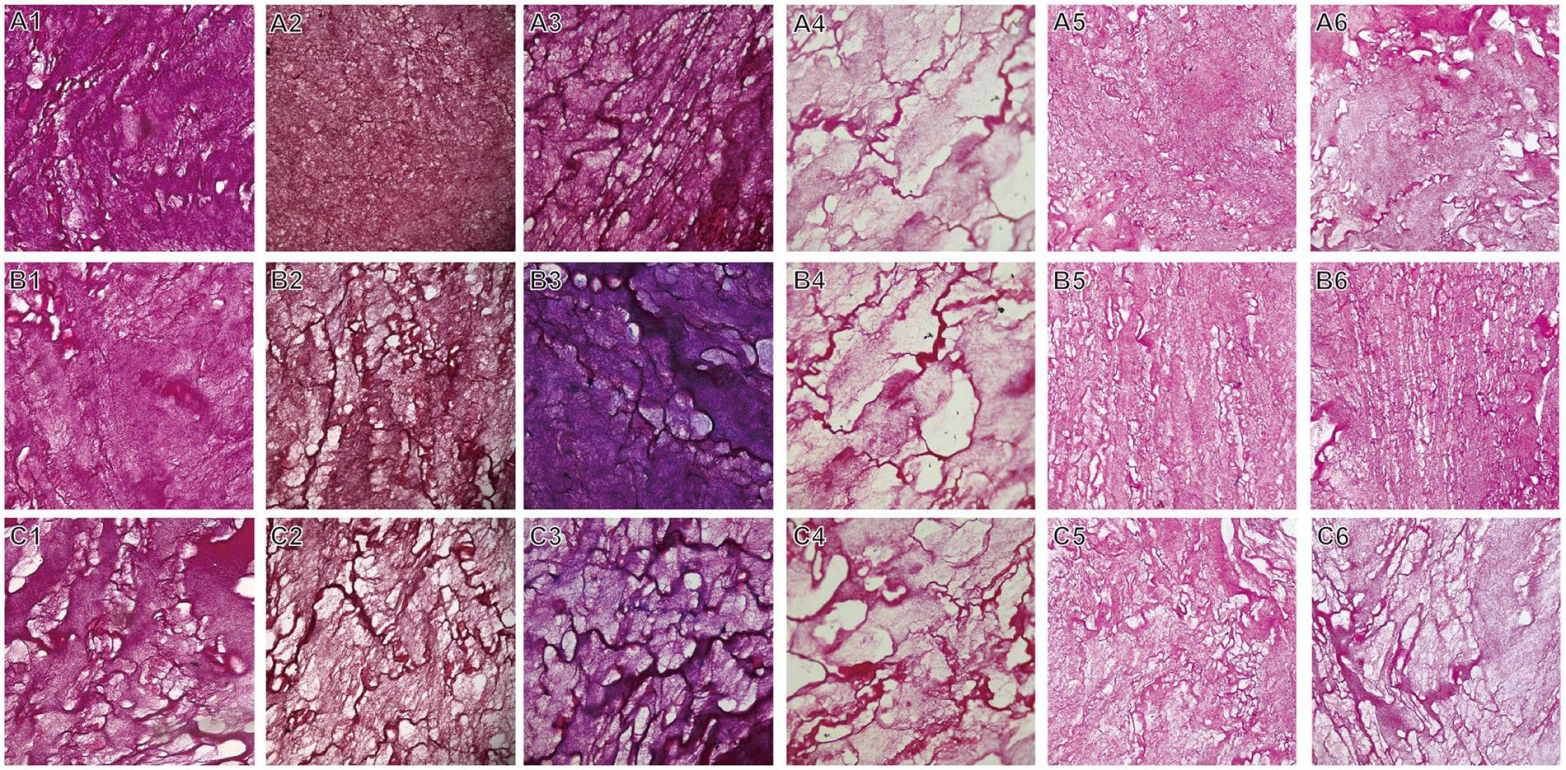
Masson’s trichrome staining and histological analyses of three segments of rabbit platelet-rich fibrin (PRF) gel. Sections from the red blood cell end (A1–6), middle segment (B1–6) and serum end (C1–6) of the PRF show decreasing compactness and increasing porosity from the red blood cell end to the serum end.

Each type of cytokine in rabbit PRF clots was quantified using ELISAs (Fig. 1A–C). The levels of PDGF-BB, TGF-β1 and insulin-like growth factor 1 (IGF-1) were significantly greater in clot1 (i.e., the RBC end of the PRF clot) than in serum1 (serum obtained during the first centrifugation of whole blood) or extract1 (supernatant harvested during the second centrifugation of the PRF gel). On average, PDGF-BB, TGF-β1 and IGF-1 levels were 3.7-, 4.1- and 3.3-fold higher, respectively, in clot1 than in serum1, but these levels were similar between serum1 and extract1. In all six rabbits, Masson staining revealed fibrin network structures that varied in gradient porosity from 34.28% ± 10.42% to 39.59% ± 10.67% and 45.53% ± 8.91% (Fig. 2A–C for all six specimens). This finding implies that cytokines were concentrated in the fibrin network of the PRF clot, especially in the region of lowest and smallest porosity (in clot1, defined as the essence of PRF, or ePRF, in a previous work^16^, thus leaving a low level of cytokines in the remaining serum1 and in extract1, which was obtained after the second centrifugation). These observations prove conclusively that the fibrin structure of the PRF clot, which is the main entity currently used in regenerative medicine, is important and necessary.

### Quantification of Cytokine Levels in Human PRFs

**Figure 3:**
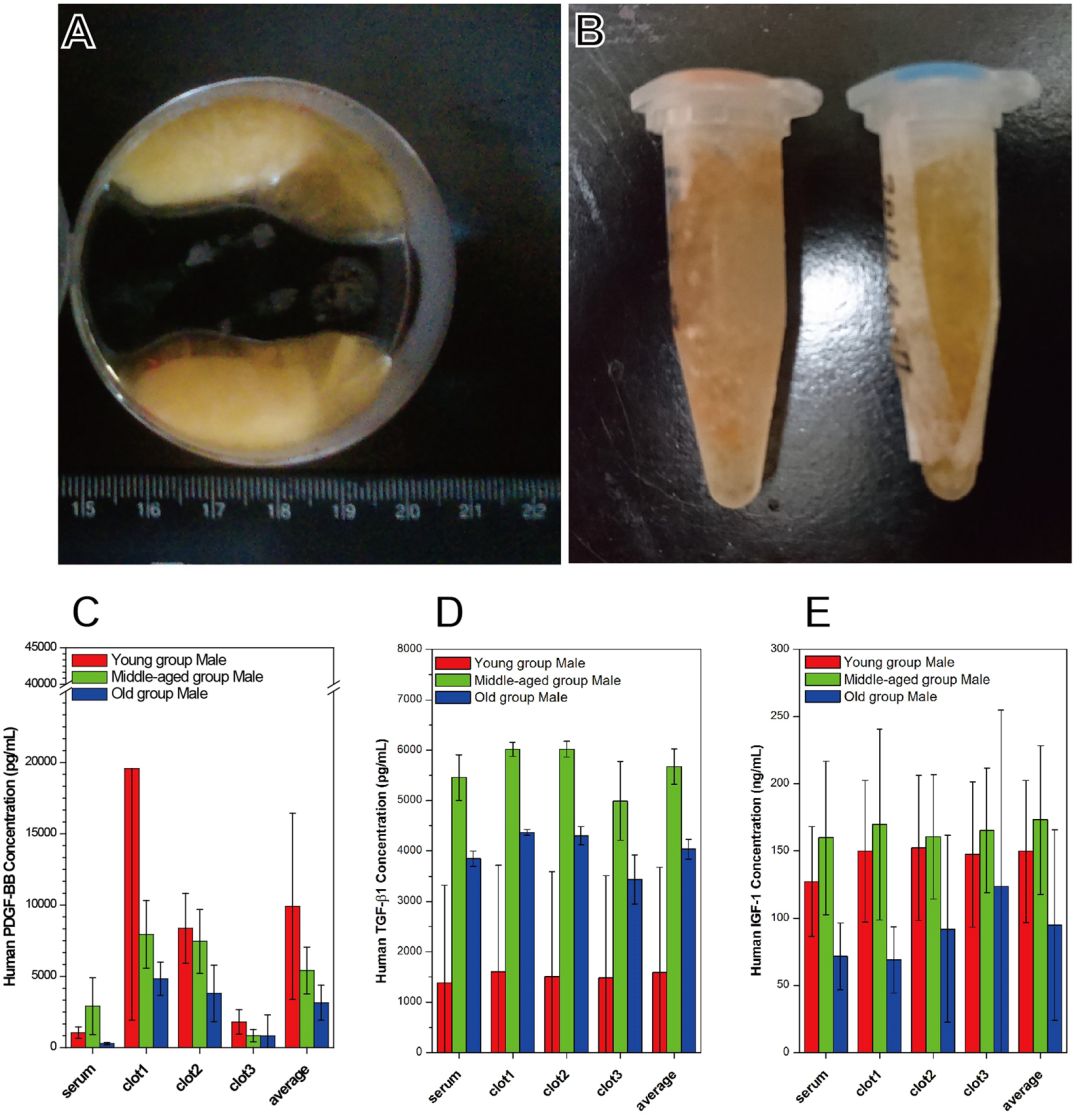
Human study: Cytokine levels in platelet-rich fibrin (PRF) specimens from men in three age groups. All PRF gels were divided into three sections: clot1 (red blood cell end), clot2 (middle segment) and clot3 (serum end). The average is across all three clots.

**Figure 4:**
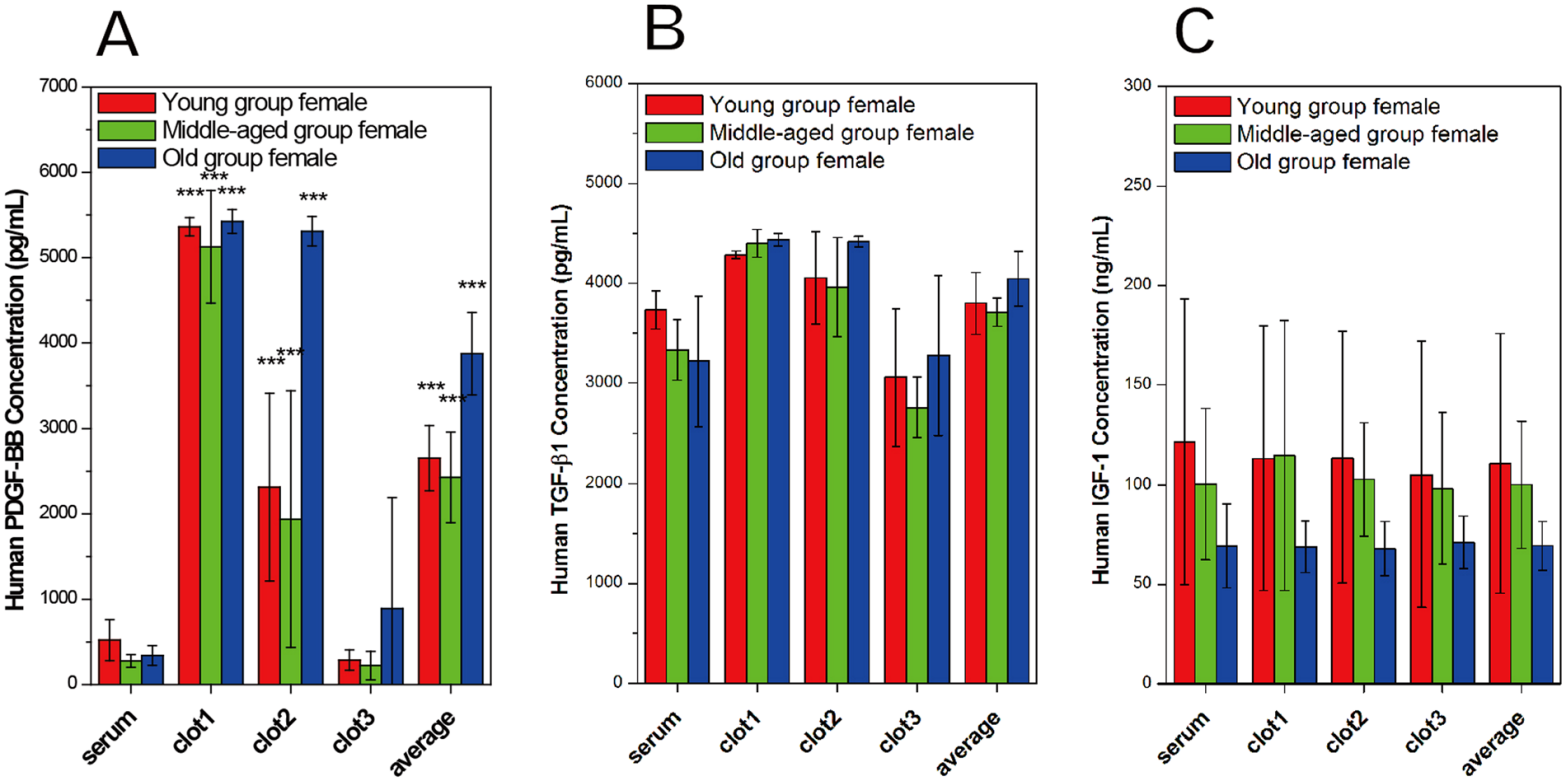
Human study: Cytokine levels in platelet-rich fibrin (PRF) specimens from women in three age groups. All PRF gels were divided into three sections: clot1 (red blood cell end), clot2 (middle segment) and clot3 (serum end). The average is across all three clots.

A total of 36 participants (18 men, 18 women) met the enrollment criteria and provided blood samples. The method of cytokine quantification was the same as that applied in the rabbit study. Figure 3A,B show a typical human PRF clot and serum, respectively, harvested after whole blood centrifugation. Compared to PRF clots obtained from rabbit blood, these were much smaller in volume and were more yellowish in color. Figure 3C–E and Fig. 4A–C show the age-specific cytokine levels for men and women, respectively. Although significant differences in PDGF-BB, TGF-β1 and IGF-1 levels were found between these groups, both PDGF-BB and TGF-β1 levels were significantly higher in clot1 than in clot2, clot3 or serum. In contrast, IGF-1 levels were similar between the clots and sera, as observed in the rabbit model. These results suggest that clot1 might be the best choice for clinical use compared to clot2, clot3 and serum because it has higher levels of PDGF-BB and TGF-β1, not because it has a near equal level of IGF-1.

A close examination of each subgroup revealed that all measured cytokines were more abundant in men than in women with one exception: TGF-β1 levels were higher in the youngest women. Both PDGF-BB and TGF-β1 levels decreased from clot1 to clot3 regardless of age or sex. On the other hand, IGF-1 levels were uniform throughout the PRF clot. Subgroup results strongly suggest that clot1 is preferable to clot3 because cytokine levels were even lower in clot3 than in serum for some groups (e.g., middle-aged men, young women and middle-aged women). Moreover, in men, cytokine levels seemed to be much more significantly influenced by age. The level of PDGF-BB in clot1 was highest in young men (i.e., 2.5- to 3.8-fold higher than the level in any other group). The opposite results were found for TGF-β1. Middle-aged and older men had lower levels of PDGF-BB and higher levels of TGF-β1 in clot1 than young men.

### Histological Analysis of Human PRFs

**Figure 5:**
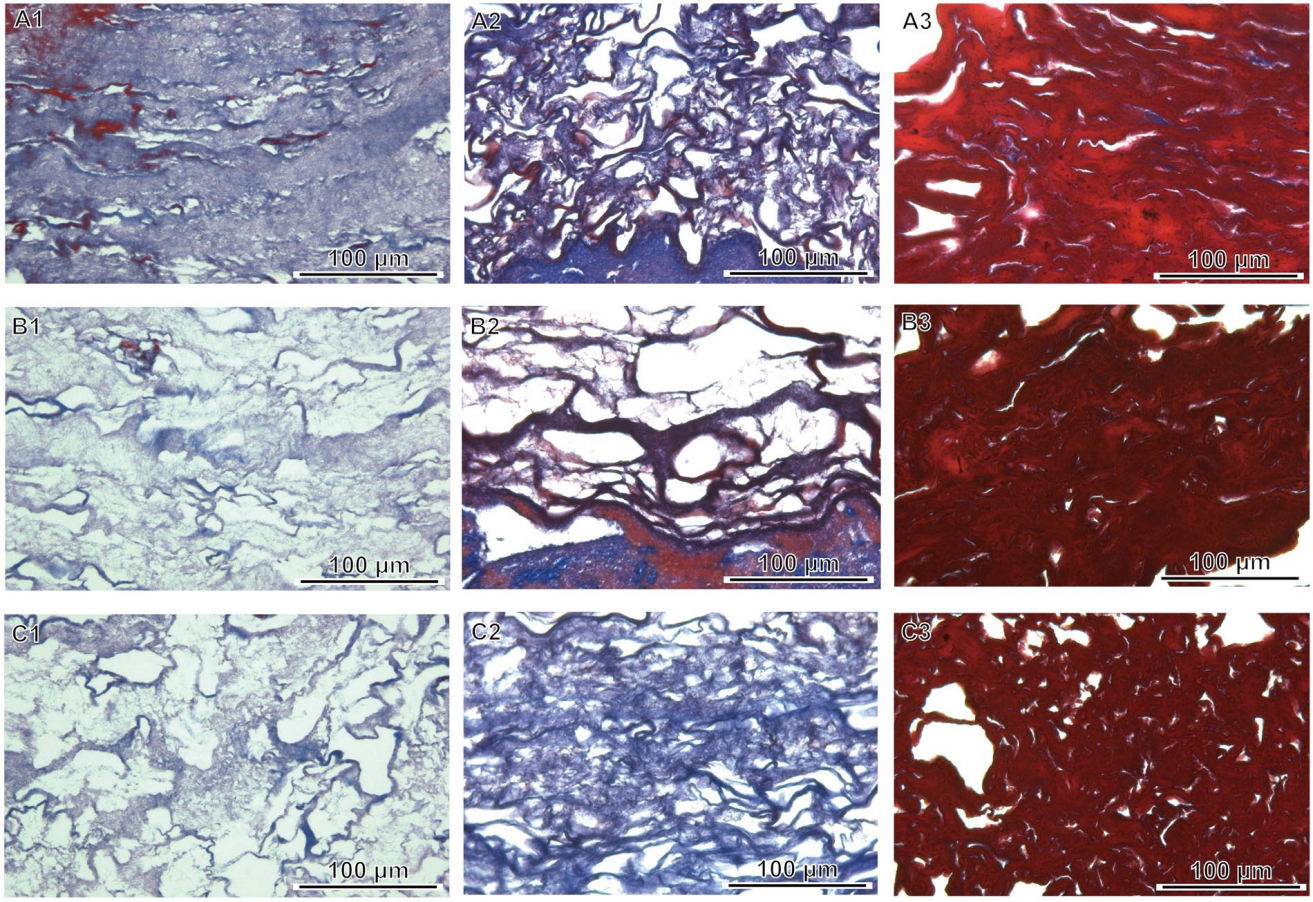
Histological analyses and Masson’s trichrome staining of three segments obtained from human PRF clots. The sections from the red blood cell end (A1–3), middle segment (B1–3) and serum end (C1–3) exhibit decreasing compactness and increasing porosity.

The stained sections of PRF clot1 (the RBC end), clot2 (the middle segment) and clot3 (the serum end) were assessed in men. The Masson trichrome-stained sections of clot1, clot2 and clot3 from young men (Fig. 5A–C) revealed that clot1 had a more compact/dense fibrin network than clot2 or clot3 (Fig. 5A) and more entrapped platelets than clot3 (the fibrin network closest to the serum layer; Fig. 5C1,C2 and C3). The levels of cytokines and the numbers of entrapped platelets had similar gradients of distribution in the PRF clot. This is not surprising given that platelets are cytokine reservoirs. This relationship also depended on age-related changes in fibrin structure and fibril size (Fig. 5). Therefore, a high platelet density or thrombin level increases the rate of fibrinopeptide cleavage and the simultaneous creation of many branch-points, giving rise to a network of thin fibers when it exceeds the rate of lateral aggregation of fibrinogen monomers. In contrast, a low concentration of thrombin slows the rate of fibrinopeptide cleavage below the rate of lateral aggregation of fibrinogen monomers, thereby resulting in the generation of fibrin fibers with thicker diameters. Porosity is therefore inversely related to fibrin diameter. A statistical analysis of PRF porosity in all human participants is shown in Table 1. A clear gradient of porosity across the PRF clot is seen in all young and middle-aged individuals but not in older individuals.

**Table 1:** Porosity Variations in Histological Biopsies of Human Blood-Derived PRF Gels.

Porosity (%) Sample	Male (*n* = 18)			Female (*n* = 18)		
	Young age	Middle age	Old age	Young age	Middle age	Old age
clot1	5.7	39.9	39.4	7.7	16.8	10.1
clot2	22.6	41.0	48.1	10.0	25.7	13.4
clot3	49.7	50.6	41.0	12.7	26.2	11.8

## Discussion

This study confirms the necessity of comparing cytokine levels in PRF gels with those in its exudates harvested after a second centrifugation (i.e., PRF extract). In the rabbit study, cytokine levels were 3 to 4 times higher in the PRF gel than in its exudates; therefore, a second centrifugation had no prominent effect on concentrating the cytokines in the exudates. The successful harvest of injectable PRF (i-PRF) by centrifuging at 700 rpm (60 *g*) for 3 min reportedly yielded a liquid PRF with the ability to release growth factors for up to 10 days^19^. Quantification and comparisons of cytokine levels were not mentioned, and regenerative bioactivity was unclear. Moreover, this protocol, which uses a relatively low centrifugation spin speed and time, requires mixing the i-PRF with bone granules or small pieces of PRF membrane debris to generate a sticky paste for clinical therapy procedures, such as bone grafts or dental implants. Based on previous results, we believe the entire PRF (i.e., the fibrin clot and material extruded from the clot) is crucial for the observed clinical efficacy. In a previous study^16^, we proposed that fibrinogen and thrombin concentrations at the RBC end of a PRF sample might be greater because of the force of centrifugation, entrapping more platelets and resulting in the generation of fibrins with smaller diameters. These smaller fibrin fibers would pack more tightly, lowering porosity and thus trapping more platelets than other parts of the PRF gel. Histological evidence for this proposal is shown in Fig. 5. However, platelets possess granules that contain clotting mediators such as factor V, factor VIII, fibrinogen, fibronectin, platelet-derived growth factor, adenosine triphosphate (ATP) and calcium ions, which are released in response to platelet activation stimuli. They also store ATP and adenosine diphosphate, which are secreted in response to thrombotic stimuli. Therefore, a firefly luciferase assay was used to determine ATP levels in the various parts of the PRF clot based on the following reaction:

$$\begin{array}{c}Luciferin+O2+ATP\to Luciferase\to Oxyluciferin+CO2+AMP+Diphosphate\\ +\,Bioluminescent\,Light\end{array}$$

**Figure 6:**
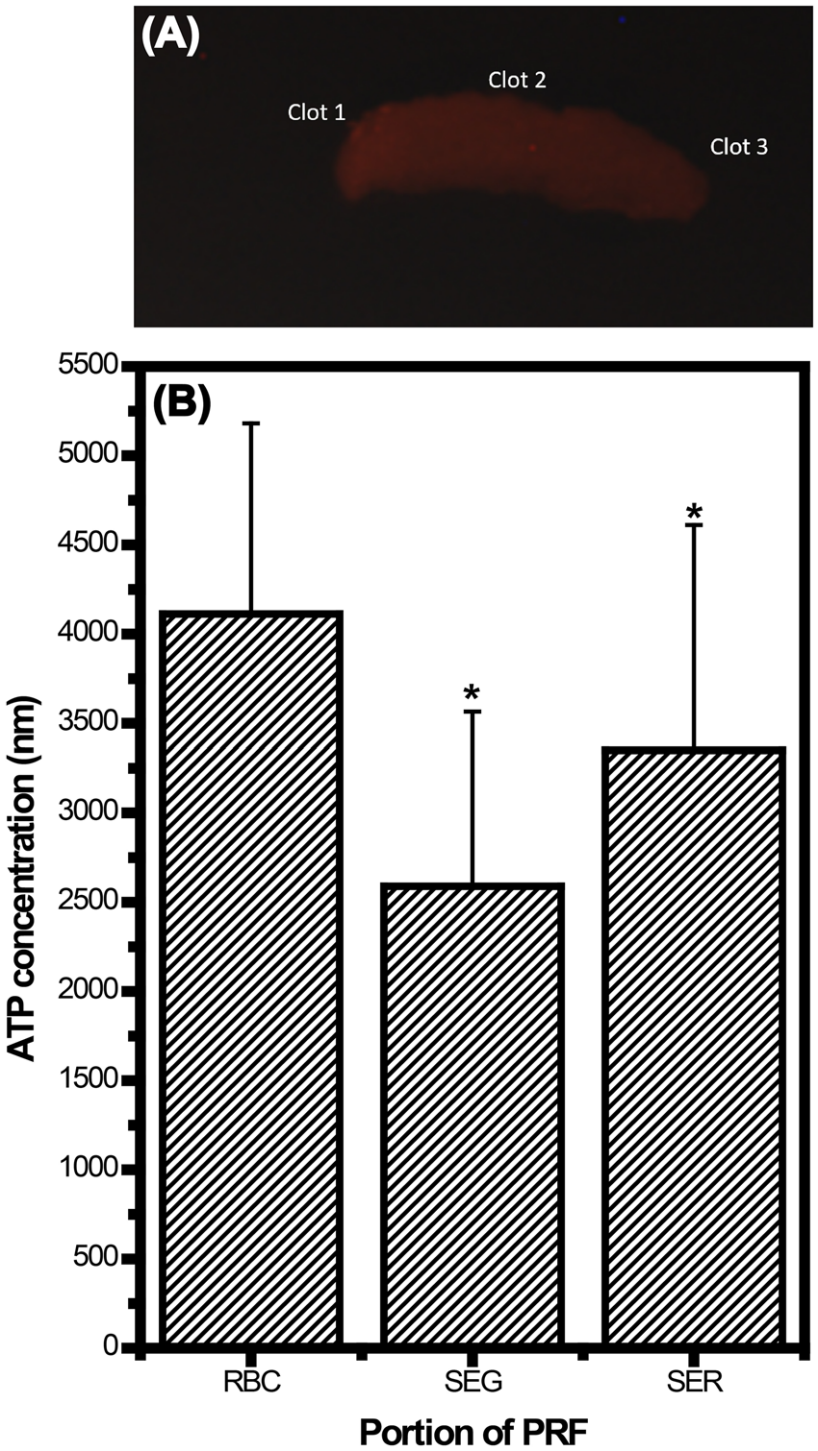
Determination of adenosine triphosphate (ATP) for as-received platelet-rich fibrin (PRF) gels: (**A**) bioluminescence image acquired from a whole PRF gel (as indicated by the red color) and (**B**) quantitative ATP for the clot1, clot2 and clot3 portions of PRF gels indicated in (**A**). Statistical results were obtained from 14 specimens of PRF gel (harvested from 5 rabbits). Results reveal that the amounts of entrapped platelets are not uniform in PRF gels, and the entrapped platelets were activated, a status that can initiate cytokine release and regeneration.

Theoretically, greater bioluminescence indicates more activated trapped platelets. Quantification of ATP and bioluminescence imaging (Fig. 6A,B) showed that the ATP level is graded across the entire PRF clot. These results prove the hypothesis; thus, the RBC end of a PRF clot is defined as the ePRF relative to various other PRF derivative products. This evidence proves that the entrapped platelets were activated, and only the activated platelets could release those bioactive species, such as cytokines, that induce regeneration in tissues. This is one reason why clinical efficacy varies case by case in patients who receive traditional courses of PRP treatment; the platelets might not have been activated in some cases if improper handling procedures were used.

All cytokine levels were significantly higher in men than in women. Notably, PDGF-BB showed the sharpest distribution gradient and the greatest levels in young men compared to other groups. This finding can be attributed to the greater regeneration ability of this group over other groups. In young men, PDGF-BB has a molecular weight and size that allows it to be easily entrapped by the fibrin network. This cytokine distribution gradient becomes less apparent when the cytokine molecular weight is smaller, as with TGF-β1 (25 kDa) and IGF-1 (7 kDa), because retention by the fibrin matrix is less likely at lower molecular weights. This pattern of cytokine distribution was observed in all groups, but the steepness of the gradient varied with molecular size. Consequently, TGF-β1 levels were not age-dependent in women and were comparable to those found in men. Evidence from the literature shows that TGF-β1 plays an important defensive role in disease pathogenesis^20^. This could be one reason why women live longer than men. Though unclear, the reasons for this gender difference could be genetic, hormonal, or environmental. For example, high levels of TGF-β1 in women might protect them against multiple sclerosis^21^.

The distributions of PDGF-BB and TGF-β1, but not IGF-1, in human PRF gel were graded, and their concentrations were much higher in PRF gel than in serum. These preliminary results, confirmed in both men and women, can be explained by a combination of factors, including: (1) extrinsic factors related to the fibrin gel structure, such as the gel porosity and the fibrin diameter, which vary from the RBC end to the serum end and (2) intrinsic factors, such as the molecular weights and sizes of the various cytokines. All cytokines are soluble and therefore should concentrate in the serum after centrifugation. However, a process akin to netting fish was observed and proven by histological biopsies, which showed many platelets trapped in the RBC end of the clot (the most compact area of the fibrin network). Therefore, a large amount of PDGF-BB was detected in the RBC end. In addition, TGF-β1^22^, a protein identified in platelets, was found in high levels at the same PRF location. Because of its much lower molecular weight^23^ and because it is produced primarily in the liver, IGF-1 is less likely to be trapped and retained in the fibrin matrix and is more likely to circulate in the blood to promote growth. Therefore, the lowest levels of IGF-1 were seen in older individuals who are expected to have much lower regenerative abilities^24^. Although PRF was first reported by Dohan *et al*. in 2006^17^, who showed that it concentrates a number of cytokines, anticoagulants and bovine thrombin are not required during preparation. In the PRF, cytokine distribution, on the whole, is thought to be the result of platelet cytokines remaining trapped in the fibrin meshes, and probably even in the fibrin polymers. This study, however, demonstrates that cytokine levels and porosity in PRF change gradually from the RBC end to the plasma end of a clot, and this change is influenced by sex and age. It is our belief that these important findings can renovate the standard clinical use of PRF and could support healthcare providers by offering improved treatment efficacy and more individualised therapy.

There are few limitations to this study. This study was designed with a small sample size. However, this study was a pilot trial used to verify results found in previous animal models; thus, the sample size is rational based on The Ethical Guidelines for Statistical Practice. Although all healthy participants in this study met the strict selection criteria, the cytokine levels we observed could be influenced by factors other than age and sex, such as genetic, hormonal, and environmental factors, could not be fully eliminated in this pilot trial.

In conclusion, this is the first systematic study to evaluate cytokine distribution in human PRF gels and its relationship to age and sex. Use of the portion that represents ePRF is necessary, and cytokine distribution in the PRF gel is not uniform. These revolutionary concepts were validated by demonstrating that: (1) cytokine levels in PRF gels after a second centrifugation are similar to those in naturally-generated blood serum and (2) the uneven porosity of the PRF network causes differences in the extent of platelet accumulation, resulting in cytokine gradients, especially for those found in platelets, such as PDGF-BB and TGF-β1. In short, this non-uniform cytokine distribution is strongly affected by the fibrin network, which is a platelet-interdependent factor, and the platelets are further associated with age and sex. Therefore, we found that cytokine levels differed with age and sex. Of course, there could be genetic, hormonal, or environmental reasons for sex- and age-related differences in cytokine levels^25–28^.
